# Distinguishing between linear and exponential cell growth during the division cycle: Single-cell studies, cell-culture studies, and the object of cell-cycle research

**DOI:** 10.1186/1742-4682-3-10

**Published:** 2006-02-23

**Authors:** Stephen Cooper

**Affiliations:** 1 Department of Microbiology and Immunology, University of Michigan Medical School, Ann Arbor, Michigan 48109-0620, USA

## Abstract

**Background:**

Two approaches to understanding growth during the cell cycle are single-cell studies, where growth during the cell cycle of a single cell is measured, and cell-culture studies, where growth during the cell cycle of a large number of cells as an aggregate is analyzed. Mitchison has proposed that single-cell studies, because they show variations in cell growth patterns, are more suitable for understanding cell growth during the cell cycle, and should be preferred over culture studies. Specifically, Mitchison argues that one can glean the cellular growth pattern by microscopically observing single cells during the division cycle. In contrast to Mitchison's viewpoint, it is argued here that the biological laws underlying cell growth are not to be found in single-cell studies. The cellular growth law can and should be understood by studying cells as an aggregate.

**Results:**

The purpose or objective of cell cycle analysis is presented and discussed. These ideas are applied to the controversy between proponents of linear growth as a possible growth pattern during the cell cycle and the proponents of exponential growth during the cell cycle. Differential (pulse) and integral (single cell) experiments are compared with regard to cell cycle analysis and it is concluded that pulse-labeling approaches are preferred over microscopic examination of cell growth for distinguishing between linear and exponential growth patterns. Even more to the point, aggregate experiments are to be preferred to single-cell studies.

**Conclusion:**

The logical consistency of exponential growth – integrating and accounting for biochemistry, cell biology, and rigorous experimental analysis – leads to the conclusion that proposals of linear growth are the result of experimental perturbations and measurement limitations. It is proposed that the universal pattern of cell growth during the cell cycle is exponential.

## Introduction

In a recent paper Mitchison [1] proposed that single cell analysis is preferred for determining the pattern of cell growth or size increase during the cell cycle. Mitchison argues that population analysis tends to average data and thus obscure the variability observed amongst individual cells. Mitchison suggests that "... they provide extra information that is not available from studies of cell populations. Without them a cell biologist can be misled."

Here I argue to the contrary, that single cell studies are more misleading than population studies. Understanding cell growth should be based on cell culture behavior rather than single cell studies. It is also argued that single-cell studies do not statistically distinguish between linear and exponential growth patterns. In contrast, pulse-labeling experiments of cultures are able to distinguish these different growth patterns. The conclusion of Mitchison [1], that linear cell growth is a valid description of cell growth during the division cycle, is reexamined here. It is shown that both the experimental data and our understanding of cell growth support exponential growth rather than linear growth.

### Purpose of cell cycle studies

As a starting point for understanding cell cycle studies, consider DNA replication. An *a priori *answer to "What is the pattern of the rate of DNA replication along a strand of DNA?" would be "the rate of DNA replication is constant." Even without any experimental measurements, our knowledge of the simple structure of DNA, varying in composition only over relatively short distances (i.e., variation in the presence of C-G and A-T pairs in the DNA sequence), would suggest that once DNA synthesis started at some origin of replication, the progress of the replication fork along the parental DNA strand would be constant. No detailed results demonstrating the constancy of DNA replication rate have appeared at a fine structure level, although there is some experimental support for a constant rate of DNA replication in bacteria [2,3]. Yet even these bacterial results are not sufficient to exclude deviations from a constant rate of DNA replication. For example, replication might start slow and speed up or vice versa.

If either of these deviant patterns – slow start with an increase in rate or rapid start and a decrease in rate – came from some experimental measurement, we would then look at the mechanism of replication and try to understand how the rate might vary; what cellular components, or properties of DNA, might regulate the rate at which DNA polymerase acts? And we would also look at the experimental evidence and critically analyze the data and methods to ensure that the experiment was valid. Our current knowledge would lead us to a critical examination of any experiments that suggested a systematic variation in the DNA replication rate.

The principle used to make this proposal is that "extraordinary claims require extraordinary evidence." Not all evidence is, or should be, treated equally. One can only think back to the famous controversy about the efficacy of highly diluted chemicals, where, to paraphrase James Randi [4], it was noted that if someone said "I have a goat in my backyard," this would be accepted, but if someone said "I have a unicorn in my backyard," one would rightly be skeptical and wish to take a look. This may lead to an asymmetry in judging experiments. Thus an experiment supporting a constant rate of DNA replication would be welcomed and easily accepted while an experiment supporting a systematic variation in the rate of DNA replication would be treated with initial skepticism.

A more logical and expected deviation from constant rate might come from local variations in the composition of DNA. As more energy is required to dissociate GC bonds than AT bonds, a continuous but slightly fluctuating rate in DNA replication might be expected depending on the local GC/AT ratio. In regions of high GC content the rate would decrease slightly; in regions of high AT content the rate would increase. How would such variation relate to the initial proposal of constant rate of DNA replication? I suggest that it would not affect the initial proposal at all. Such minor variations do not impinge on the fundamental concept that once replication starts DNA replication moves along at a rate determined primarily by the nature of the replication fork components. That there might be minor variations in movement depending on variations in DNA composition does not affect the basic law of DNA replication, that the rate of DNA replication is determined by the nature of the polymerase system, and that external controls do not systematically affect the passage of the replication fork down a strand of DNA.

Taking this analysis a step further, suppose we could measure the rate of DNA replication in a single cell or within a single replicon. Imagine that such observations showed that there were individual cellular variations depending on myriad factors such as local curvature of the DNA, the concentration of cytoplasmic or nuclear components, whether the replication point is at the interior or exterior of a nucleoid or nucleus, and so on. Imagine that different cells differed in rates of replication of the same regions of DNA. If the average rate was still constant (as discussed above), this would not impinge on our statement that DNA replication, once initiated, is constant. An encyclopedic description of all the possible modes of replication in all cells is not the goal when trying to understand the law of DNA replication rate.

The point made here is that the idea of a general biological law leading to understanding biological phenomena is not subject to criticism or rejection based on minor deviations from the law, whether inherent in minor deviations of cellular structure in all cells or deviations from one cell to another cell. Studying the deviations from a general law is not the purpose of deriving a general law. In the case of DNA, the important principle is the understanding that the movement of the replication fork along the DNA is not subject to regulation but that once started DNA moves at a constant rate. This general view is not weakened by minor deviations based on AT/GC ratios, or deviations between different cells.

The object of cell cycle studies is not to know the variation in some particular process, but to understand the underlying logic of the process. What is desired is the general "law" of cell growth; and the population, as we shall see, is a better source of this pattern than an individual cell.

### Collective and individual studies of human growth

**Figure 1:**
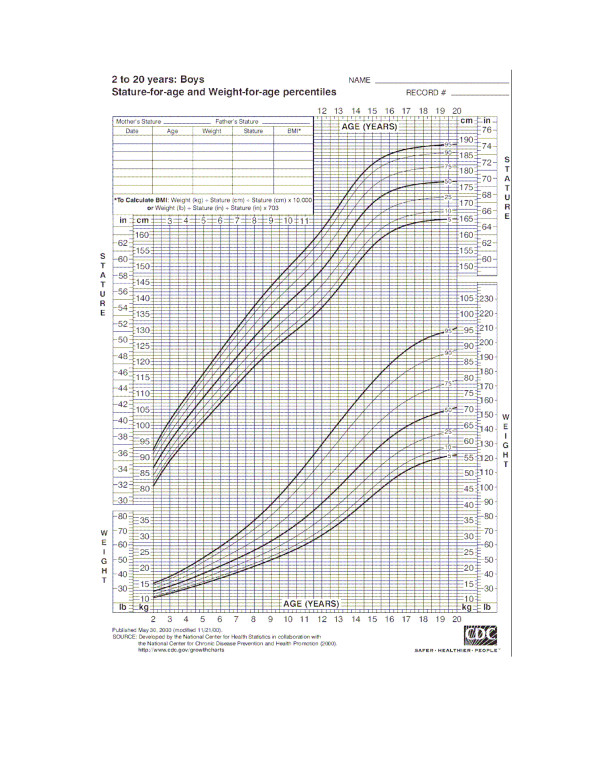
Human growth as a function of age. This chart, developed by the Center of Human Health Statistics, was obtained from a web search and shows the mean height (50^th ^percentile) and deviations from the mean height in percentiles.

As another example of deriving general growth laws from individual measurements, it is interesting to consider the example of human growth. Figure 1 shows a standard "growth chart" obtained from measurements of thousands of individuals at particular ages. The central line of the height pattern is determined from measurements of thousands of individuals. It is possible, and even probable, that not one single individual fits this particular curve. An individual may be above the curve at some times and then be below the curve at other times. There is individuality in height during human growth, but this does not prevent one presenting the description of human growth as the average of many patterns.

**Figure 2:**
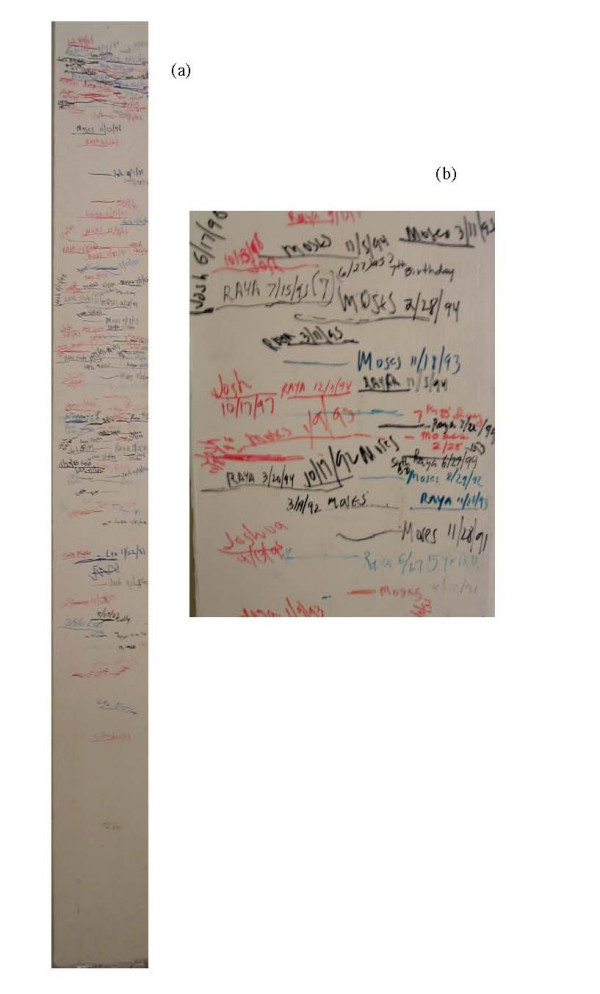
Growth board for two individuals. This board has been used to record over the last 19 years the heights of Raya Cooper and Moses Cooper. At various times the child would stand against the board and their height would be measured by drawing a line and dating the line. At the left is the full board and at the right is a close-up of a portion of the board.

**Figure 3:**
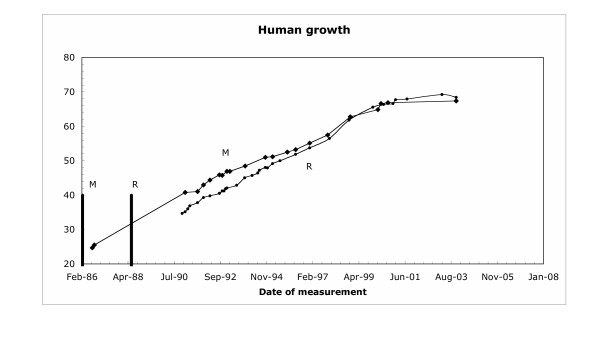
Chart of height of two individuals. The heights from the growth board from Figure 2 were determined and the heights are plotted in inches. The vertical bars are the birth dates.

The individual results on which Figure 1 are based are not publicly available, and so to look at some individual results I must rely on my own experimental observations. I have studied the growth of my grandchildren over 19 years by noting their heights at different times. For many years I have kept a "measuring board" upon which I recorded the height of my grandchildren at random occasions, usually family gatherings. When measuring time occurs the grandchildren stand against the board, a line is drawn at the top of their head, and the date of the line is noted. The board on which some of these results are recorded is illustrated in Figure 2. The results for two of my grandchildren are plotted in Figure 3. Note that there is no standard, simple, pattern of growth. Sometimes experimental error leads to the finding that two measurements over some period of time are the same, suggesting that perhaps there was no growth during that period. Experimental error is very likely an explanation of these deviant observations. Yet these individual observations do not prevent us from proposing and accepting the standard growth pattern as illustrated in Figure 1.

As we shall see below, this "apparent" cessation of human growth has clear resonances in subsequent analyses of single cells of both *E. coli *and *S. pombe*.

The growth pattern of the population is the important result, not the individual growth patterns. One does not want to walk around with an encyclopedic description of all the patterns observed for all individuals. That is not the object of growth studies of human beings. And it should not be the object of cellular growth studies. What is desired is the general "law" of cell growth, and the population, as we shall see, is a better source of this pattern than an individual cell.

### Exponential is the expected pattern of cell growth

What is the expected pattern of cell growth during the division cycle? The overwhelming majority of a cell's mass is the cytoplasm; i.e., all that is not cell surface or cell genome. The cytoplasm is the amorphous content of the cell composed of ribosomes, enzymes, ions, water and soluble components. For eukaryotes one can even include mitochondria as part of the cytoplasm. During cell growth this cytoplasm produces more cytoplasm. As there is no expectation that cytoplasm produced by a cell cannot immediately enter into biosynthesis, this means that mass increase (i.e., cytoplasmic mass increase) is exponential. Consider a newborn cell of size 1.0. During the first interval of growth the cell would make 0.1 units of cell mass so that at the end of the interval the mass is 1.1 units. During the next interval of growth both the original and new cytoplasm would produce cytoplasm so that the additional cytoplasm appearing during the second interval would be 0.11 units of mass, leading to the cell size being 1.21 units after two growth periods. With successive increases in cell mass the pattern of mass increase would be observed to be exponential. Just as with compound interest in a bank where money accumulates exponentially because money added to the initial principal generates interest during later time periods, the cell mass would be expected to increase exponentially.

In contrast, linear growth means that in any interval the amount of mass added to the initial cell is constant. Thus, in the first interval 0.1 units would be added, in the second 0.1 units again, and so on. The difference between exponential and linear growth is that with exponential growth the absolute increase in cell mass increases during the division cycle, but with linear growth the absolute increase in cell mass is constant during the division cycle.

### Problems with linear growth

There are two problems associated with linear growth. The main *a priori *problem is that as the cell gets larger, the cytoplasm becomes steadily more inefficient. Inefficiency is defined as a cell producing less mass per unit extant mass compared to more efficient use of the extant mass. Mass increase would be efficient when extant cell mass makes new mass as fast as possible. As a cell grows, more cytoplasm is present, and efficiency considerations alone would suggest that the rate of addition of new mass would continuously increase (i.e., exponential growth).

With linear growth the extra cytoplasm does not increase the absolute rate of cell mass synthesis. In essence, the new cytoplasm does not make new mass. With linear growth the relative rate of mass increase (i.e., mass synthesis per extant mass) decreases, which means that the ribosomes, after some growth, are not working as efficiently as before. One can imagine two models for the reduced efficiency of ribosomes: (a) not all ribosomes are active in protein synthesis or (b) ribosomes each work at decreasing efficiency.

The second, correlated problem may be even more important, as linear growth requires a jump or saltation at some time during the division cycle, either in the middle of the cycle for proposed bi-linear patterns, or at division for pure linear patterns during the cell cycle. It is unavoidable that linear growth requires sudden increases in the pattern of biosynthesis of the cell. Thus, a cell that grows adding 0.1 unit of mass at each time interval would do this continuously for one cell cycle. At the instant of division the two daughter cells together would now begin to add 0.2 units of mass each time interval. There would be a sudden increase in the rate of mass increase at the instant of division or at some particular time during the cell cycle.

One could imagine all sorts of mechanisms to solve this problem, and many mechanisms have been proposed. One could imagine that new sites of uptake made during the cell cycle are activated only at division. Or perhaps new ribosomes and protein synthetic elements are not activated until division or at some time during the cell cycle. The problem with these proposals (and here we remain in the realm of supposition) is that there is no known mechanism to accomplish linear synthesis. One might show up soon, but at the moment this is a major problem.

Again, as in the discussion of DNA replication above, the proposal of linear growth is an extraordinary claim (as witnessed by Mitchison's "surprise" [1] when he came to the linear conclusion, as he may have expected exponential growth), and such claims requires "extraordinary evidence." As we shall see, there is no such "extraordinary" evidence. As we shall further see, the evidence actually supports exponential growth rather than linear growth.

### Pulse or differential analysis vs. integral analysis

**Figure 4:**
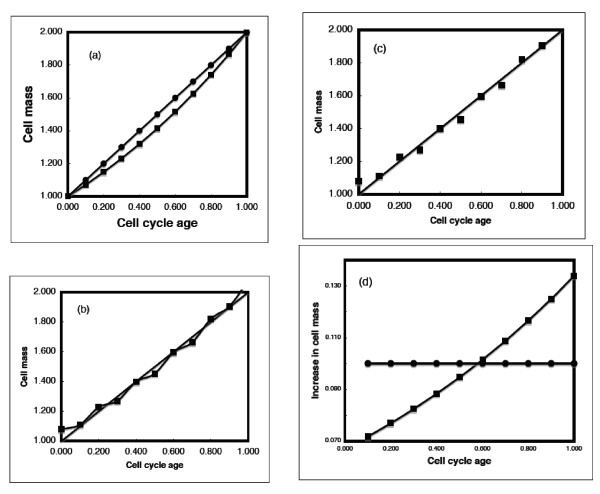
Comparison of experimental measurements of exponential and linear growth. (a) Plotting of exponential and linear growth over one doubling shows that the lines are quite similar (circles, linear; squares, exponential). (b) Adding of small variations up and down to alternate exponential points shows that the lines for exponential and linear growth are very similar. (c) Removing the connecting line and looking at only the data for the varied exponential line shows that one cannot eliminate exponential growth by a straight line on rectangular coordinates. (d) Comparison of differential measurements of growth showing that one can distinguish between exponential growth and linear growth using differential measurements.

Consider the experimental problem in determining the pattern of cell growth using single-cell observations. The main problem in distinguishing between linear and exponential growth is that when plotted as the amount of mass present at any moment, the two graphs are quite comparable (Figure 4a). As shown in Figure 4a, over a doubling in mass (i.e., one cell cycle), the difference between linear and exponential growth is quite small. This is more clearly seen if one considers errors of measurement as shown in Figures 4b and 4c. If one gives a small amount of variation to measurements of exponential growth, and then plots this along with a straight line (Figure 4c), it can appear to the eye that the data fit a linear pattern. The point of Figures 4a,b,c is that by merely watching a cell grow through the cell cycle it is very difficult, and perhaps impossible, to distinguish between linear and exponential growth.

Rather than using overall cell growth as one does with microscopic examination, it is of interest to consider the differential approach. The differential of an exponential pattern is exponential, while the differential of a linear pattern is constant. This is clearly illustrated in Figure 4d where the difference between the lines is obvious.

In a differential experiment one would measure the change in cell size or cell mass using some radiological method that indicates the change over a short time period. If one had a synchronized culture and measured the incorporation of some isotopic label that measured cytoplasm increase, the incorporation pattern would be constant if growth were linear, whereas there would be an increase in incorporation over the division cycle if growth were exponential.

The arguments presented by Mitchison [1] are based on the assumption that one has a good method to measure cell mass using microscopy. While length measurements entail no need to standardize length measurements, the use of optical methods to measure mass is fraught with problems. There is no proof that such methods are independently able to measure cell mass accurately. Early measurements are inherently suspect because there is no external standard by which to judge the accuracy of the method. There is no known set of cells that can be used to standardize the optical mass measurements. In addition, there is some experimental error in each of these microscopic size measurements, whether length or cell mass is being determined. These experimental variations will greatly affect the ability to distinguish, or rather the inability to distinguish, exponential from linear growth.

### Linear growth models

The linear growth model has a long history. Using interferometry on single cells, Mitchison proposed linear growth in dry mass in fission yeast [5-8] and in budding yeast [5,7-9]. The same technique was also used on *Streptococcus *[10], where declining rate curves were found. Kubitschek [11-19] also proposed linear growth of bacteria based on studies of cell size on synchronized cultures. Conlon and Raff [20] have also proposed that the mass of eukaryotic cells increases linearly.

### *Escherichia coli *growth during the cell cycle

The analysis *Escherichia coli *growth illustrates many of the ideas and problems presented above. In one of the earliest studies, microscopic analysis indicated that *E. coli *growth was exponential [21]. A more accurate differential approach with microscopic studies of cells was performed by Ecker and Kokaisl [22]. They pulse labeled growing cells, fixed them, and analyzed incorporation in individual cells by autoradiography. They observed that larger cells incorporated more amino acids and uridine than smaller cells, a major step toward supporting exponential growth.

**Figure 5:**
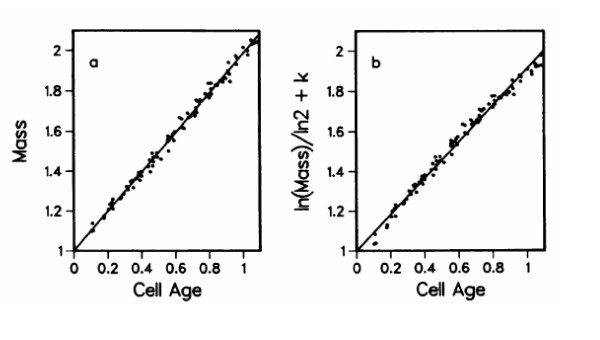
Reanalysis of the data of Kubitschek [16]. At the left is the original data of Kubitschek and at the right is a replotting on logarithmic coordinates. The details are presented in the text.

It is interesting to consider results on *E. coli *related to the proposal of linear growth, particularly in light of the discussion of difficulties in distinguishing linear from exponential growth. Kubitschek [16] proposed that the accumulation of mass during the division cycle of *E. coli *is linear. This proposal was made on the basis of size measurements of cells that were synchronized using sucrose gradients to select the smallest cells from an exponential culture. Cell sizes were determined with an electronic cell size analyzer. As shown in Figure 5, Kubitschek's results cannot be used to distinguish between linear and exponential growth. Kubitschek [16] plotted the measured sizes of cells of different ages on a rectangular graph drawing the best straight line through the experimental points. He then drew a line for exponential growth that deviated visibly from these points. His statistical analysis of this type of graph indicated that the data were consistent with the proposal of linear growth and excluded exponential growth. The exponential line tested was not the best fit to the data but was determined by only two datum points. A reanalysis of the published data of Kubitschek on a semilogarithmic plot [23] is also shown in Figure 5. Without going into the details of the analysis, the conclusion resulting from the analysis in Figure 5 is that one cannot distinguish between linear and exponential growth using these data. Thus, the size measurements of Kubitschek [16] are compatible with an exponential rate of synthesis during the division cycle [23]. Any deviations as noted are extremely slight in terms of the differences in cell size measured with a Coulter Counter.

**Figure 6:**
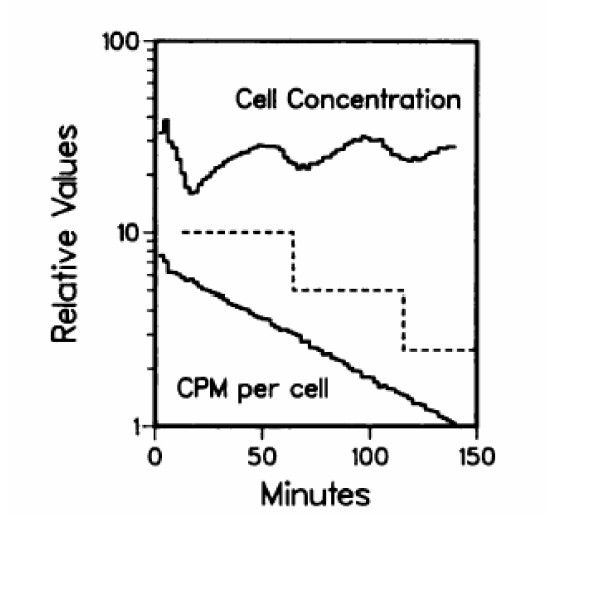
Cell cycle analysis of leucine uptake (and protein synthesis) during the division cycle. A100-ml amount of *E. coli *B/r lys mutant cells in culture medium (10^8 ^cells per ml growing in minimal medium with glycerol and lysine) was labeled for 2 min with 2 uCi of [14C]leucine (450 mCi/mmol; New England Nuclear Corp.). The cells were then filtered, washed, and analyzed by assaying the radioactivity per cell eluted from the membrane-elution apparatus. The dashed line is the expected pattern for a constant rate of leucine uptake and protein synthesis during the division cycle. This constant rate is predicted by a model of linear rate of increase in mass during the division cycle. The upper cell elution curve has oscillations that are due to the initial cell age distribution of the cells at the time they were filtered. The decrease in the dashed line is placed at the end of the first division cycle as indicated by the cell elution curve. The decreasing exponential curve of radioactivity per cell indicates exponential growth.

The most conclusive and convincing demonstration of exponential growth in *Escherichia coli *comes from a differential experiment using membrane elution that does not perturb cells [23]. Cells growing in steady state, exponential, growth were pulse-labeled with an amino acid and then bound to a membrane. Newborn cells eluted from the membrane were counted and the radioactivity per cell was determined. The results clearly indicate an exponential pattern of incorporation (Figure 6). If incorporation were linear then the step pattern illustrated by the dotted line in Figure 6 would be found. The exponential decrease in counts per cell during elution is precisely what is expected for exponential incorporation of amino acids. Conversely, the evidence presented in this membrane-elution analysis does not support the fundamental data on which the linear model for increase in mass was derived, that is, the constant uptake of molecules during the division cycle of bacteria [24].

It is important to understand why membrane-elution is a valid experiment. First and foremost, the membrane-elution method has been used to obtain the DNA pattern of synthesis during the *E. coli *division cycle, and this result has been supported by an enormous amount of additional experimentation. As one example, the membrane-elution results have explained both the increase in DNA content with growth rate [25-27], and the DNA contents gave the first accurate measurement of the size of the *E. coli *genome [25]. This model of DNA replication, with bilinear DNA (not mass) synthesis at particular growth rates, has been supported by myriad experiments. Thus, the cells bound to the membrane divide in order as required by the method. Further, the labeling is performed prior to any binding to the membrane, so there is no perturbation of the cells. The use of the membrane-elution method has been discussed extensively along with the details of this experiment and others [28].

In the history of the study of the growth of *E. coli *there is one result that should be noted, that of Hoffman and Frank [29] who performed early time-lapse studies of bacterial growth. They observed a single cell that appeared to stop growing for a few minutes. This result was, and remains singular, and is reminiscent of the duplicate points in the individual human growth curves in Figure 3. But this singular result cannot, and should not, be used to say that cells stop growing at a certain point. That is because this result is not a replicable and repeatable result.

### The correct *Escherichia coli *growth law

The growth law of *E. coli *is essentially exponential, but in reality is more complicated than the simple exponential growth pattern presented above. The growth law is so close to exponential that it is essentially indistinguishable from this simple mathematical pattern. The growth of a cell is the sum of the growth or biosynthesis of its individual components. Thus, if one knew all of the growth patterns of the individual components, the growth law would be the weighted sum of these growth patterns. As the cytoplasm is by far the major component, the other parts of the cell do not contribute measurably to the growth pattern of the whole cell. It is of interest to explore this "real" growth law for *Escherichia coli *as the synthetic patterns of the major components of the cell are well known, as is the cellular composition.

The uptake of molecules is exponential for precursors of protein [23], stepwise for precursors of DNA [3,27,28,30-38], exponential for precursors of RNA [39-41], and complex but almost exponential for precursors of peptidoglycan and cell membrane [42-47].

**Figure 7:**
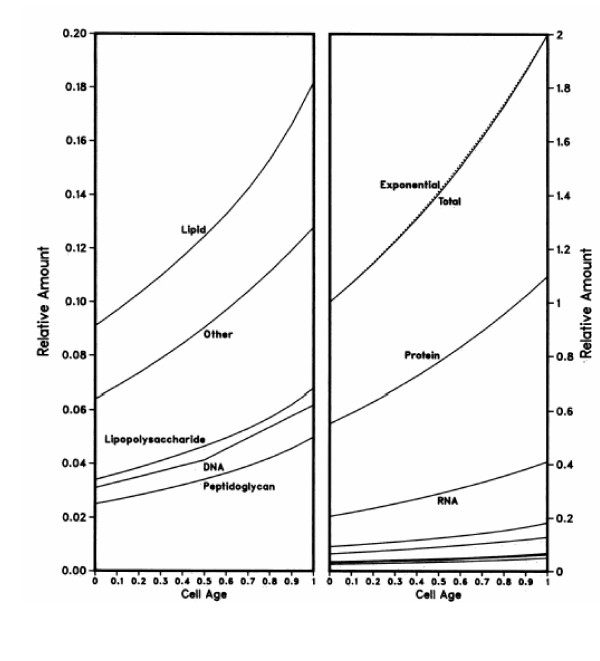
Biosynthesis rates of the various components of the bacterial cell during the division cycle [48]. The curves are drawn proportional to their relative contributions to the cell, using the results of Neidhardt [54] for *E. coli *grown in minimal glucose medium. The percentages of dry weight are as follows: peptidoglycan, 2.5%; DNA, 3.1%; lipopolysaccharide, 3.4%; other (including polyamines, salts, glycogen, etc.), 6.4%; lipid, 9.1%; RNA, 20.5%; and protein, 55.0%. The RNA, protein, and other materials were assumed to have an exponential increase. The synthesis rates of lipid, lipopolysaccharide, and peptidoglycan were presumed to be proportional to the peptidoglycan synthesis rate [42, 44-47]. The rate for DNA synthesis was assumed to be linear with a doubling in rate in the middle of the division cycle [3, 25, 27]. The dotted line is an exponential increase; it indicates the difference between the calculated mass increase and exponential mass increase. The two panels are the same graphs but differ in scale to illustrate the biosynthesis rates of the less prominent material.

When an accounting is made for each of the cellular components, and the weighted patterns are used to obtain the total exponential growth law as a sum of the individual growth patterns of the individual cellular components [48], the results are presented in Figure 7. It is clear that while there are minor deviations from a true exponential pattern, the actual result of the individual growth components is that growth is essentially exponential during the cell cycle.

### Analysis of yeast growth during the cell cycle

Mitchison has been proposing aspects of linear growth for over four decades [1]. This idea stems mostly from Mitchison's early work on gas exchange and his proposal of a rate change point (RCP) in the cell cycle.

Without going into the entire history of the yeast growth studies, it is interesting to point out one instance where there is a direct confrontation of the linear and exponential proposals using the same experimental data. What is most fascinating about the paper by Mitchison [1] analyzed here is that in this paper Mitchison does not discuss this clear contrast in conclusions based on a common set of experimental results.

**Figure 8:**
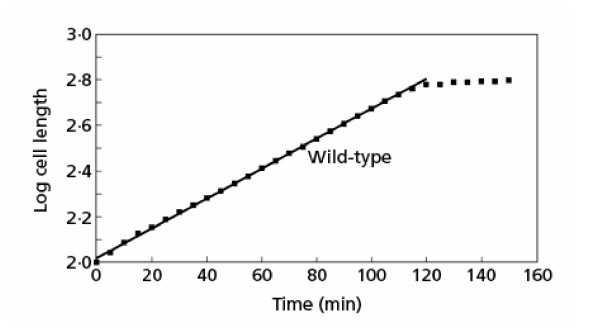
Growth in length of a single wild-type cell of *S. pombe*. The data for the cell lengths from Figure 2 of Sveiczer *et al*. [49] are plotted on a semi-logarithmic scale. The data are indicated by the filled squares. The straight line drawn through the points is the best fit based on a minimization of deviation of points from the straight line. A straight line on semi-logarithmic coordinates indicates exponential growth.

The original data on *S. pombe *cell-size measurements made by Mitchison and his associates were kindly sent to me by e-mail by Dr. Bela Novak. The original data of Sveiczer *et al*. [49] were replotted using semi-logarithmic coordinates (Figure 8). Linear coordinates, used in the original publication, give an upwardly curving line that may appear, to the eye, two comprise two linear segments. [Note: In theory, length may not be a precise measure of cell mass, as one must also assume that the diameter is constant. For the sake of clarity of argument, it is accepted here that cell length of *S. pombe *is a measure of cell mass.] As shown in Figure 8, the data for the wild-type *S. pombe *fit an exponential growth pattern well. There is no need to invoke any change in growth pattern, nor is there any deviation from exponential until the end of the cycle. I used linear regression analysis to compare the different models. The comparisons listed in Table 1 are from the original publication of a debate over this issue [50], where the *r*2 values for different analyses are presented. An *r*2 value of 1 means a perfect fit, and the higher the value the better the fit. Values above 0.9900 are essentially perfect fits to the data and are for all practical purposes indistinguishable. When the first 11 points (before the proposed RCP) are analyzed for a linear fit, a good fit to a linear regression is obtained (case A), and the same is found for the second linear segment of 13 points after the RCP (case B). Since in each of these examples two parameters are required for each segment (an origin and a slope for each line), the total number of parameters to get a fit to all the data is four.

**Table 1:** Statistical comparison of linear and exponential models (from [49]). Cases A–C were linear regressions of the original data on rectangular coordinates; case D was a linear regression of the logarithm of the original data, so that the fit to an exponential case was tested.

**Case**	**Points analysed (no. of parameters)**	**Segment analysed**	***r*2 value**
A	11 (two)	First linear segment	0.99850
B	13 (two)	Second linear segment	0.99888
C	24 (three)	Two linear segment spline	0.99959
D	24 (two)	Single exponential	0.99935

If a best fit to two linear segments with a single bilinear spline fit is analyzed (case C), we find a good fit as well, although in this case there are three parameters to the formula. These three parameters are the common midpoint value between the two linear segments, and the two slopes of the linear segments.

An analysis using all 24 points in the two proposed linear segments and fitting them to a single exponential model gives an essentially indistinguishable fit (case D), although in this case there are only two parameters in the exponential model, a single origin and a single slope. Observe that the statistical fit for the two-parameter exponential model (case D) is even better than the fit to both two two-parameter linear models (cases A and B).

How does one distinguish between the different models? The numerical distinctions (*r*2 values) between the different models are negligible. Therefore it is best to use the simplest model and this is obviously case D, where only two parameters are needed to fit all of the data. That the statistical differences between the models in Table 1 are negligible can be seen if one considers that a model with 46 parameters, taking each point as the start of a line segment, and having a slope going perfectly to the next point, would yield an *r*2 value of 1.0000. Yet this model with a perfect fit would be excluded as being too complicated and arbitrary because of the large number of parameters used to get this perfect fit. Simplicity considerations – Occam's Razor – suggest that the two-parameter model that accounts with a single formula for all of the points is to be preferred over more complex models (i.e., models with more parameters). The visual indication that growth is exponential is supported by the more precise statistical analysis (Table 1). The conclusion from this analysis is that growth of yeast during the cell cycle is exponential, consistent with the basic molecular biological ideas regarding mass growth during the division cycle.

**Figure 9:**
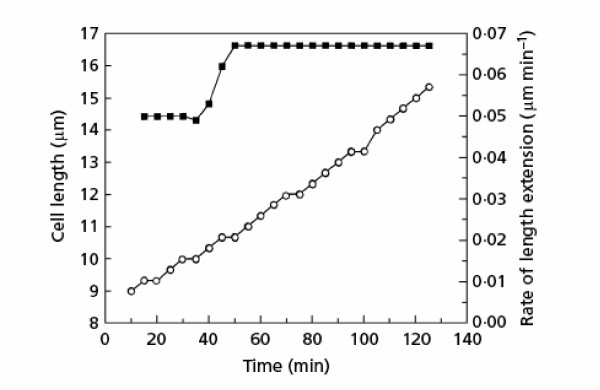
Length extension in the growing period of a single wild-type cell of *Schizosaccharomyces pombe*. Lower curve, cell length; upper curve, smooth rate of extension (see text). Data from article by Mitchison, Sveiczer and Novak [51]. See text for detailed analysis.

Akos Sveiczer (pers. comm.) has drawn my attention to a rebuttal of this conclusion by Mitchison, Sveiczer and Novak [51] who presented an analysis of a single cell of *Schizosaccharomyces pombe*. Their results are shown in Figure 8. The relevant text related to this figure is:

The linear regression on a semi-logarithmic plot used by Cooper is not sufficiently sensitive, so we have used the much more sensitive measure of the rates of length growth. The difference between successive length measurements was taken from the unsmoothed data and these differences were then smoothed by the 'rsmooth' command of the Minitab program. One result is given in Fig. 1 [original paper figure number; here it is Figure 9] with the length measurements and the smoothed rates. The rate pattern is clearly one that would be given by two linear segments with a rate change of about 30%, though the sharpness of the step rise will be somewhat diminished by the smoothing process. It is quite different from exponential growth where the rate should increase steadily throughout the growth period. So here is a cell which certainly does not grow exponentially. In other cells which we have examined, the pattern is less clear. There is a step at the RCP but there may also be other rate changes before and after this point which vary with the exact points at which the growth period starts and stops. These are not regular in their appearance and pattern, and occur because of the high sensitivity of the analysis on data that are limited by slight changes in focus and by limited resolution of the optics and of the measurements on projected photographic images. This degree of variation makes it impossible to use a formal statistical test between two simple models of linear versus exponential growth. However, we have seen no cell showing simple exponential growth. Estimation of the RCP by eye is surprisingly effective since the eye carries on a smoothing process over minor changes. It is worth mentioning that the growth curves for wee1 mutants have a much more conspicuous interphase rate change of 100% and no rate patterns. It seems most unlikely that the elimination of the wee1 gene product causes a change from exponential interphase growth to two linear segments.

This analysis illustrates and supports, in bold outline, the points and conclusions made in this paper. A careful reading of these ideas indicates the problematic nature of the data supporting linear growth of *S. pombe*. Note that Mitchison, Sveiczer, and Novak present the data for a single cell [51], and note that other cells that they have observed have different patterns and that they have not seen an exponential pattern in any of these other cells. It is as though one were to criticize the human growth chart (Figure 1) using the data for individuals (Figure 3). But even a cursory look at the data shows more problems. From my perspective the data fit an exponential curve as well as any curve (see Figure 4a). But note that the first point has a length value of 9 (presumably the newborn cell) and the data end at length of approximately 15. If this cell were a normal cell, representative of all cells, the length would double over one doubling time and the graph should end around length 18. This discrepancy suggests that the length growth of this particular single cell was constrained by the growth conditions (lying on an agar surface, not being free to show full extension as would occur in liquid growth) and thus one should be skeptical of this result. Regarding the deeper analysis of the differential graph (upper curve, Figure 9) it can only be noted that the extensive smoothing program used eliminated the slight variation at 90–100 minutes. One can only ask: why not just take the data as is and propose that at some point during the cell cycle the cell ceases to grow rapidly and stops for a moment? This is the true reading of this single cell result, and one can only ask why this result is not presented as a "growth law".

Again, as with human growth curves (Figure 3) and *E. coli*, there is a piece of data saying that growth ceases for a moment (Figure 9). But it is clear that this is not a reproducible result and in fact is eliminated in the smoothing analysis in Figure 9. This discarding of abnormal results indicates a belief, even evidenced by Mitchison, Sveiczer and Novak, that such aberrant results should not be made part of the proposed growth law.

While it may be that "beauty is in the eye of the beholder", scientific conclusions should not be made using such subjective criteria. The statement that "Estimation of the RCP by eye is surprisingly effective since the eye carries on a smoothing process over minor changes..." certainly needs proof before it can be accepted. To my eye, the data of Sveiczer, Novak, and Mitchison [49] suggest exponential growth; to them linear with an occasional RCP. It is for this reason that one must be skeptical of the microscopic determination of cell growth patterns.

The irreproducibility of the experimental results (e.g., Figure 9) is shown in the statements: "... In other cells which we have examined, the pattern is less clear.... but there may also be other rate changes before and after this point which vary with the exact points at which the growth period starts and stops... These are not regular in their appearance and pattern, and occur because of the high sensitivity of the analysis on data that are limited by slight changes in focus and by limited resolution of the optics and of the measurements on projected photographic images...." [51].

This means that the data have been selected and the reader does not know which experiments have been discarded and which reported. Let us assume that we had all the data, from numerous measurements on cells, and we saw that there was a lot of scatter and problematic readings. This type of result, not reported, could be used to criticize the reported results. It is not proper merely to produce those particular single-cell results that fit a preconceived idea. One must present all of the experiments done whether or not one thinks they are valid. Only then can a reader judge the validity of the published data.

The discussion of the *wee *mutants is also of interest. I can only assume that the statement that there are "...no rate patterns" means that when the differential is analyzed the points are all over the place. This, of course is to be expected, as with a small cell the microscope resolution problems would lead to greater errors in observation. That, of course, is the key problem with single cell microscopic measurements, that one can get results that are related to a single cell and not related to a general "growth law."

It is legitimate to propose that there is no general growth law to be discovered or proposed, even for a particular cell line. However, the assumption made in this paper, and used for the analysis of the data on *S. pombe*, is that there is a growth law, and that it is up to good, reproducible experiments to discover that growth law.

I suggest that the yeast cell grows exponentially during the division cycle. One may believe, if one wishes, the more complex RCP model. However, in order to do this one must note that the data fit an alternative model equally well. The alternative model is simpler, and the theoretical analysis of cellular growth is strongly consistent with the exponential growth model. The exponential growth model is based on a very simple and biochemically sound explanation for exponential growth.

The data on yeast cell growth and biochemistry presented here are strongly supportive of exponential growth between divisions. No rate changes between linear growth segments need be postulated as controlling elements in the cell cycle. The data fit the proposed pattern of exponential mass synthesis during the division cycle. There is no reason to accept the linear model of cell growth during the division cycle of *S. pombe*. The data fit an exponential pattern for cell growth during the division cycle.

Mitchison participated in a public debate involving this issue [50,51]. It is worth reviewing this published analysis in detail to see that the experimental data clearly support exponential growth during the cell cycle.

[N. B. In the Mitchison paper that was the impetus for this analysis [1], the two figures in the paper, although neither is specifically referred to by name or number in the paper, have been accidentally switched. Figure 2 should be associated with the legend of Figure 1 and Figure 1 should be associated with the legend of Figure 2.]

### Growth of mammalian cells during the division cycle

Conlon and Raff [20] have proposed a linear growth model for mammalian cells. This conclusion has been shown to be incorrectly drawn as it is based on incorrect assumptions, incorrect logic, and errors in experimentation [52].

For example, consider one of the experiments used by Conlon and Raff to demonstrate linear cellular growth during the division cycle. Conlon and Raff studied cells cultured in 1% fetal calf serum, forskolin and aphidicolin. Aphidicolin inhibits DNA synthesis. While mass increased, there was no concomitant increase in DNA. The cells were incubated for 216 hours (9 days). The cell volume was measured using a Coulter Counter, although in one experiment total protein content was measured. Conlon and Raff realized that it is extremely difficult to distinguish linear from exponential growth over one doubling time. Therefore they measured mass increase over a longer period of time (approximately 3 or more normal interdivision times). The problem with this experiment is that inhibition of DNA synthesis does not allow an exponential increase in cell number. Therefore the experiment is subject to the critique that aphidicolin inhibition produced the observed results. The results do not reflect the situation in normal, uninhibited and unperturbed cells. For example, there could have been exponential growth during the first "virtual cell cycle". Thereafter the limitations of DNA content would lead to the observed linearity of growth as measured over the extended period of analysis. But this linearity should not be taken to indicate that cell mass increases linearly during the normal cell cycle.

Even if cells grow linearly during the division cycle, if the rate of mass increase is measured over a number of cell cycles with uninhibited cells, then *a priori *there should be evidence of an approach to exponential mass increase. If the rate of mass increase during the first cycle is 1.0, during the second cycle it should be 2.0, during the third cycle 4.0, and so on. Thus, even on its own terms, with linear mass increase during the division cycle, the experiments of Conlon and Raff [20] on the pattern of mass increase are flawed by the presence of an inhibitor of DNA synthesis. Suffice it to say that the reader should compare the original proposal of Conlon and Raff [20] with the published critique and analysis of this work [52], and see that exponential growth is clearly an acceptable and superior model for mammalian cell growth during the division cycle.

Mitchison [1] has resurrected some early data of Prescott [53] on the growth of *Amoeba proteus *and showed these data in Figure 2 of his paper (caption beside Figure 1, as noted above). In this figure the growth of cells indicates a downward curvature indicating, according to Mitchison, the antithesis of exponential growth. But these experiments are subject to the critique that the method itself (Cartesian diver balance) can affect cell growth. What is not shown in the figure is the growth of cells after division; if it were true that newborn cells change their growth rate at division, there would be a sudden change in growth rate.

### The importance of knowing the growth law

One could argue that it makes no difference what the growth law is, as the cells will double in size every cell cycle no matter what the growth law, and steady-state growth can occur in either case. But the difference between linear and exponential modes of cell cycle growth is whether specific mechanisms exist that need to be looked for and understood to produce linear growth, or whether there are no further specific mechanisms to regulate cell growth. If growth is exponential, no further search for growth control is needed as exponential cytoplasm growth can explain the total cellular growth. If, however, growth is linear then there must be a mechanism, as yet undiscovered, that can give linear growth.

It is of interest to consider one particular mechanism off-handedly proposed by Mitchison [1], that has an allure as an explanation of abrupt changes in growth consistent with linear growth patterns. This is the involvement of "gene doubling" at replication of a particular part of the genome, or even a doubling in the total genome, that would lead to a doubling in the rate of growth. A. Sveiczer (pers. comm.) also proposed this. Let us consider the simple case that there was a rate limitation imposed by the presence of a single copy of a particular gene that was suddenly relieved by the replicative doubling of this gene. According to Mitchison, this gene doubling could lead to a doubling in the rate of growth. But let us say that at a particular point in the division cycle a particular gene product is now made at twice the rate as before gene doubling. Since the major force in mass growth is the activity of the protein synthesizing system, this would mean that in some way the gene product that has now begun to be made at twice the rate now leads to a jump in the rate of protein synthesis of the protein synthesizing system. This is hard to visualize, as one would expect that as more proteins are made from the gene, there would only be an extremely gradual and probably imperceptible change in the rate of accumulation of protein synthesizing activity. That is because the ribosomal activity would not suddenly double. One would not expect any sudden jump in any cellular component that would lead to the jump in the rate of cell growth that would explain linear growth. Until this proposed mechanism of gene doubling is rigorously explained and described, it cannot be used to support the proposal of linear growth or bi-linear growth during the division cycle. Of course it is very likely that even at the doubling of a gene there are other limiting factors and there is in fact no change in the rate of synthesis of the product made from the genes that doubled.

### General growth laws and specific growth laws

Throughout this paper it has been assumed that there is *a growth law *of cells that can be found and understood. It is assumed that cells do not willy-nilly choose this or that growth pattern depending on the whim of the moment. If one wished to propose that cells have variant growth laws, and different cells of the same clone do different things during the division cycle, one is free to propose that idea. I cannot imagine any support for that idea. But it is clear is that there is now an explicit statement of the idea that there is a general law that is independent of single-cell observations. Any argument to the contrary should now be clearly presented so that the issue is joined.

### Summary

Exponential growth during the division cycle is very likely the general growth law for all exponentially growing cells. Linear growth during the division cycle is not supported by theory or experiment. Regarding the maxim, "extraordinary claims require extraordinary evidence," it is clear that the evidence for linear growth does not meet the criterion of being extraordinary. Differential experiments are better than integral experiments, and differential experiments support exponential growth during the division cycle.

## Competing interests

The author declares that he has a definite and obvious interest, personal and scientific, in clarifying the nature of cellular growth during the division cycle. The author hopes that this paper clarifies the long-standing controversy between linear and exponential models of cell growth during the division cycle. Aside from this declared interest, the author has no financial or other competing interests.
